# Mutation Rate, Spectrum, Topology, and Context-Dependency in the DNA Mismatch Repair-Deficient *Pseudomonas fluorescens* ATCC948

**DOI:** 10.1093/gbe/evu284

**Published:** 2014-12-23

**Authors:** Hongan Long, Way Sung, Samuel F. Miller, Matthew S. Ackerman, Thomas G. Doak, Michael Lynch

**Affiliations:** ^1^Department of Biology, Indiana University, Bloomington; ^2^National Center for Genome Analysis Support, Indiana University, Bloomington

**Keywords:** neutral evolution, mutation hotspots, nonrandom mutations, phage evolution

## Abstract

High levels of genetic diversity exist among natural isolates of the bacterium *Pseudomonas fluorescens*, and are especially elevated around the replication terminus of the genome, where strain-specific genes are found. In an effort to understand the role of genetic variation in the evolution of *Pseudomonas*, we analyzed 31,106 base substitutions from 45 mutation accumulation lines of *P. fluorescens* ATCC948, naturally deficient for mismatch repair, yielding a base-substitution mutation rate of 2.34 × 10^−8^ per site per generation (SE: 0.01 × 10^−8^) and a small-insertion-deletion mutation rate of 1.65 × 10^−9^ per site per generation (SE: 0.03 × 10^−9^). We find that the spectrum of mutations in prophage regions, which often contain virulence factors and antibiotic resistance, is highly similar to that in the intergenic regions of the host genome. Our results show that the mutation rate varies around the chromosome, with the lowest mutation rate found near the origin of replication. Consistent with observations from other studies, we find that site-specific mutation rates are heavily influenced by the immediately flanking nucleotides, indicating that mutations are context dependent.

## Introduction

Mutations are a primary source of the genetic variation that is harnessed by selection to drive evolutionary processes. Although thorough estimates of the mutation rate and spectrum (defined here as the rate distribution of different types of mutations at different sites) are necessary to understand many evolutionary processes (Halligan and Keightley 2009; Lynch 2010a), our knowledge of these critical parameters is limited to a handful of model organisms. The most accurate estimates of the mutation rate and spectrum come from mutation accumulation (MA) studies, whereby replicate lines are taken through single-cell bottlenecks, allowing for the accumulation of all but the most deleterious mutations (Mukai 1964; Kibota and Lynch 1996; Halligan and Keightley 2009). In most modern analyses, after a number of generations, genome sequencing of each replicate line provides an unbiased estimate of the genomic mutation rate and spectrum in the organism.

Because mutations are rare events, most MA studies observe only a handful of mutations (Lynch 2010a; Ness et al. 2012; Sung, Ackerman, et al. 2012; Keightley et al. 2014), making it difficult to understand more complicated mutational patterns. For instance, the mutation process is influenced by multiple factors, such as the genomic position and nucleotide context (Petersheim and Turner 1983; Yakovchuk et al. 2006; Lee et al. 2012; Foster et al. 2013); the mutation rate is higher close to the replication terminus and lower near the origin of replication, clearly indicating that mutations are not generated randomly at different genomic positions (Sharp et al. 1989; Foster et al. 2013). It is possible to study these processes in more detail by using DNA mismatch repair-deficient (MMR^−^) strains, in which mutation rates are usually elevated by 1–2 orders of magnitude relative to MMR^+^ strains (Oliver et al. 2000). The MMR pathway, which is conserved in both prokaryotes and eukaryotes, detects and repairs base-mismatches arising from DNA replication errors (Aquilina and Bignami 2001; Hsieh 2001; Kunkel and Erie 2005).

Here, in an effort to further understand complicated mutation processes, we conducted a 2-year MA experiment on the MMR-deficient strain *Pseudomonas fluorescens* ATCC948. We chose to study the mutation process in *P. fluorescens* for three reasons. First, *P. fluorescens* plays extremely important ecological roles in the rhizosphere (Batchelor et al. 1959; Huang et al. 1960; Cox and Adams 1985; Loper 1988; Cook et al. 1995; Shim and Yang 1999; Farhadian et al. 2008; Loper et al. 2012). Second, recent studies have shown that different strains of *P. fluorescens* are highly diverse at the genomic level (as is true for many bacterial species, see Lukjancenko et al. 2010); the core genes shared by ten *P. fluorescens* strains represent only 45–52% of the total proteome of each strain (Loper et al. 2012). The remaining genes, defined as accessory genes, are absent in one or more of the ten strains (Silby et al. 2009; Loper et al. 2012). Although core genes cluster around the origin of replication, accessory genes segregate toward the replication terminus, raising the question whether this genomic architecture results from differential mutation pressure, genome-wide differences in natural selection or other factors such as conjugation/recombination. In principle, the elevated divergence in gene content near the terminus could be a result of relaxed purifying selection on genes in these regions. Finally, the abundant prophage sequences present in the *P. fluorescens* genome (Loper et al. 2012) provide an opportunity to study the unexplored mutation patterns of prophages.

## Materials and Methods

### Strains and Cultures

The *P. fluorescens* strain ATCC948 was obtained from the American Type Culture Collection. Nutrient broth (EMD Chemicals, Gobbstown, NJ) was used for liquid culturing of MA lines for DNA extraction and freezing at −80 °C (final glycerol concentration: 10%). Nutrient agar (Becton, Dickinson and Company, Sparks, MD) was used as solid media for the MA transfers. The type strain SBW25 was kindly provided by the Vaughn Cooper lab, University of New Hampshire. In the *mutS* locus of ATCC948, a 41-bp insertion caused *mutS* dysfunction (5′-ACTGGTGCCGCGCCCCAGGCCAGGGACAGGCCGTCGAAGGT-3′), and the *mutS* gene was amplified (forward primer 3F: 5′-CGGGAAAAGATGCTCTATGAAG-3′, reverse primer 2R: 5′-AGCACGGTCAGTTCGAAATAGT-3′), and sequenced by bidirectional Sanger sequencing (see Sanger sequencing detail below).

### Fluctuation Tests and Mutation Rate Estimation with Reporter Construct

Cells from a single colony were inoculated into 4 ml nutrient broth with 11 replicates for each subject cell line and incubated for 48 h on a tissue rotator at 30 °C. Cell density was estimated from colony forming units (CFU) on nutrient agar plates (1.68 × 10^8^ cells/ml for p20 final MA line (generation 5240—with spontaneously recovered *mutS* function); 2.24 × 10^8^ cells/ml for p20 at generation 328—with the ancestral dysfunctional *mutS* caused by the 41-bp insertion; 1.72 × 10^8^ cells/ml for the original ATCC948 culture; 4.79 × 10^9^ cells/ml for SBW25). Cell cultures (2 ml for the two p20 lines; 500 µl for the original ATCC948 line and SBW25 line) were then centrifuged, resuspended into 100 µl, and plated onto nutrient agar with 50 µg/ml rifampicin (Dot Scientific, Burton, MI) and incubated at 30 °C for 2 days. There were on average 4.36 rif-resistant colonies for the p20 final MA line, 2,051.64 mutant colonies for the p20 line at generation 328, as well as 656.00 for the original ATCC948 culture and 56.36 for SBW25.

Mutation rates from the fluctuation tests were calculated using the Ma–Sandri–Sarkar Maximum Likelihood Estimator method in FALCOR (Hall et al. 2009).

### MA Transfers and Genome Sequencing

All 45 MA lines originated from a single *P. fluorescens* ATCC948 cell. For each bottleneck of the MA lines, cells from a single colony were streaked onto nutrient agar plates and incubated at 24 °C. Each growth cycle represents an average of 19.92 generations and 2–3 days. Colonies which were closest to a marker line were picked to avoid a bias to bigger colonies. Growth rate of cells was estimated every month by CFU. The entire experiment took 710 days, and 263 transfers, and each MA line reached an average 5,240 generations.

Genomic DNA was extracted and purified using the Wizard Genomic DNA Purification Kit (Promega, Madison, WI). DNA libraries for Illumina sequencing with an insert size of 350 bp were constructed by the Center for Genomics and Bioinformatics, Indiana University, Bloomington. Paired-end sequencing was done by BGI (Shenzhen, China) with a mean sequencing depth of 82.72× and 99% of all MA lines’ genomes were covered (supplementary table S1, Supplementary Material online).

### De Novo Assembly and Annotation of the *P. fluorescens* ATCC948 Genome

SOAPdenovo2 (Luo et al. 2012) was used for de novo assembly of the *P. fluorescens* ATCC948 genome for the major mutation analyses, using paired-end reads from five randomly selected MA lines (p1, p2, p67, p73, and p74). After eliminating scaffolds smaller than 10 kb—to remove singletons and scaffolds contaminated with plasmid sequences—a draft genome (ATCC948-1; NCBI BioProject ID: PRJNA264995; sequence accession number: JSFL00000000) with 61 scaffolds and a total size of 5.72 Mb, GC content 60.71%, N50 143,421 bp, and 42,277 gaps, was obtained and used as the reference assembly. Paired-end reads from all 45 samples were also individually de novo assembled as above, for *mutS* sequence detection. We used five lines to assemble the ATCC948-1 genome in order to prevent individual lines’ base substitutions from being represented in the draft genome, as shared mutations are extremely rare (supplementary file S1, Supplementary Material online). However, indels for the five MA lines used for the assembly could not be resolved due to incorporation of the indels into the assembly (supplementary file S2, Supplementary Material online). Thus, it was necessary to generate a second assembly to specifically identify indels in these five MA lines (the second assembly ATCC948-2 used these randomly selected lines: p4, p32, p49, p64, p79; NCBI BioProject ID: PRJNA264995, sequence accession number: JSFM00000000). The sequence identity between the draft genomes ATCC948-1 and ATCC948-2 is 98.40% after alignment with progressiveMauve (Darling et al. 2010), so most of the analyzed sites for mutation analysis were shared. The output indels positions were then located, formatted, and oriented back to the ATCC948-1 draft genome by blasting 60 bp upstream or downstream flanking sequences from the ATCC948-2 draft genome with NCBI-BLAST-2.2.28+/BLASTn (>95% sequence identity and >54 bp alignment length).

The draft genome of ATCC948-1 was annotated with BASys (VanDomselaar et al. 2005). The total draft assembly is 91.53% predicted coding sequence (CDS). There are 7,782 predicted CDSs, with an average length of 827 bp. Three types of empirically verified start codons are used in this strain: ATG (for 46.22% of all CDSs), GTG (28.27%), TTG (25.51%) (Kim et al. 2009).

### Mutation Analyses

We followed a consensus approach for identifying base substitutions, described in detail by Denver et al. (2009), Ossowski et al. (2010), and Sung, Tucker, et al. (2012): Briefly, sorted paired-end reads with a quality score greater than 20 from 45 MA lines were aligned to the assembled genome and forward and reverse reads (three of each) were required to ensure that amplification artifacts were removed. The alignment was parsed into SAM format with SAMTOOLS (Li et al. 2009), and the overall consensus (>50% of all reads at a site across all lines) and the consensuses of individual lines (>80% of all reads at a site) were parsed out. Because the average coverage depth is so high (82.72×), we opted for a lenient criteria for consensus identification in an effort to reduce false negative mutation calls by analyzing as many sites for putative mutations as possible. Due to the high mutation rate of mutator lines, a lower criterion for overall consensus calls allows for the analysis of multihit mutations, namely, mutations occurring in different lines at the same site (Drake 2007), or mutant line with much higher depth of coverage than other MA lines at certain sites, which will reduce the overall read consensus to be well below the expected binomial distribution for reads for just a single mutation. Thus, these criteria far exceed the expectation due to sequencing error (0.39%, see below), and do not affect the vast majority of analyzed sites in all MA lines, because on average 99.53% (SE: 2.38×10^−3^%) of the mapped reads are identical to the overall consensus in all samples (supplementary table S1, Supplementary Material online).

The majority of false positive mutation calls are generated from mismapped reads, for example, paralogs were mistakenly mapped to a single locus. Previous studies have shown extremely low false positive mutation calls using the consensus method (Lynch et al. 2008; Denver et al. 2009; Ossowski et al. 2010; Lee et al. 2012; Sung, Ackerman, et al. 2012; Sung, Tucker, et al. 2012), but even then, we took extra precaution to ensure that mismapped reads were not responsible for the mutation calls: 1) We required that base substitutions be identified by two independent alignment algorithms based on the two mappers BWA v0.6.2 (Li and Durbin 2010) and NOVOCRAFT v2.08.1, in order to avoid program-specific misalignment; 2) each read was only allowed to map to one site (BWA option: sampe –n 1; NOVOCRAFT option: novoalign –r None) with mapping quality >20; and 3) mapping paralogous reads to a single locus would generate what appears to be heterozygous sites in this haploid bacterium, so sites with minor allele reads greater than 20% were filtered out before further analysis.

Mutations that passed these criteria were then called for the sample(s) with a line-specific consensus that differed from the overall consensus. With the consensus approach, we were able to analyze an average of 98.70% of the 5.72 Mb *P. fluorescens* ATCC948-1 draft genome in each of the 45 MA lines. We verified with polymerase chain reaction (PCR) and Sanger sequencing 75 base-substitution mutation calls from different MA lines and scaffolds (supplementary table S2, Supplementary Material online); the finding of no incorrect calls supports the ability of the consensus method to accurately identify mutations in MA studies.

A similar approach was used for indel analysis: Mapped paired-end reads in SAM format from two different mappers were used to call indels, requiring the support of at least three forward and three reverse reads each, as well as supporting reads from greater than 30% of total reads at the site. Reads with indels are innately more difficult to align, and are generally found in homopolymeric runs which cause alignment issues, so it is necessary to use a lower criterion to identify an individual line’s consensus. Due to the reduction in the read support requirement, we supplemented the BWA/NOVOCRAFT analyses with BreakDancer 1.1.2 (Chen et al. 2009) and Pindel 0.2.4w (Ye et al. 2009), which re-align reads by detecting indel break points by a local sequence pattern growth approach and reduce indel-induced artifacts. We randomly picked a small proportion of indels—covering most scaffolds and MA lines—to verify by Sanger sequencing, and verified all the 67 indel calls that were successfully PCR amplified (supplementary table S3, Supplementary Material online).

We also estimated sequencing error rates using all mapped sites where all lines’ consensuses were identical to the overall consensus. For each individual nucleotide type, sequencing error rates were calculated by dividing the total number of discordant base reads by the total number of analyzed reads in all lines at all sites. The sequencing error rates were 0.43% (A sites), 0.33% (C), 0.34% (G), and 0.44% (T). The pooled nucleotide sequencing error rate is 0.39%.

### Mutation Call Verification with Sanger Sequencing

We designed 95 pairs of primers for base-substitution verification (supplementary table S2, Supplementary Material online) and 96 pairs of primers for indel verification (supplementary table S3, Supplementary Material online) with BatchPrimer 3 (You et al. 2008). The primers were synthesized by Integrated DNA Technologies, Inc. (Coralville, IA), and PCR was performed using MYTAQ polymerase and buffer (Bioline, London, UK). PCR amplification was successful for 75/95 pairs of base-substitution primers and 67/96 pairs of indel primers, and the amplified target DNA was unidirectionally sequenced using an Applied Biosystems 3730 automated sequencing system at Indiana Molecular Biology Institute, Bloomington, IN. For all mutations, the mutation was verified in the mutant line and confirmed to be absent in the original ATCC948, which is from the same batch culture of the unrevived MA progenitor culture. Alignments and analysis of mutations were done with MUSCLE in MEGA5.2 (Edgar 2004; Tamura et al. 2011).

### Binning the Draft Genome for the Topology Analysis and Mutation Rate Normalization with GC Content

To determine the mutation topology, we chose arbitrary bin sizes of 50 kb and 10 kb for wavelet transformation. Foster et al. (2013) recently showed that changes in bin size do not influence the stability of wavelet transformation. We blasted each scaffold of the draft genome ATCC948-1 against the complete reference genome of *P. fluorescens* SBW25 (GenBank accession number: NC_012660.1). For SBW25, the origin of replication is at chromosome position 6721956–6722539 and the replication terminus (*dif*-like sequence) is at 3377028–3327055, predicted by DoriC 5.0 (Gao et al. 2013). Average positions of blast hits with larger than 500 bp alignment length and at least 70% sequence identity were used to order and orient the scaffolds in our draft genome. The ordered and oriented scaffolds were divided into 81 bins (origin of replication in bin 1, replication terminus around bin 41), with bin one starting and ending at the origin of replication and proceeding clockwise; each 50 kb bin contained on average 271 base substitutions; no bins spanned multiple scaffolds. Fourth-order Daubechies wavelet transformation was then conducted on the binned and normalized mutation rate (see below for normalization details) using Wolfram Mathematica v8.0.

Mutations at GC sites occur at a higher rate than those at AT sites and variation in GC content between windows could cause changes in mutation counts between bins. To calculate a normalized mutation rate for each bin, we divided the observed number of AT (U1) or GC (U2) mutations within a bin by the average mutation rate of that class within the whole genome ($\bar{\mu }1\;\text{and}\;\bar{\mu }2$). This gives us the number of nucleotides we would expect to give rise to the number of mutations observed. Then we multiplied the expected number of nucleotides by the average genomic mutation rate (µ), to give the GC normalized mutation rate (*U*), which was used to plot the topology:

$$U=\left(\frac{U1}{\bar{\mu }1}+\frac{U2}{\bar{\mu }2}\right)\times \mu .$$

### Mutation Context Analysis

“Context” refers to the single 5′ and 3′ flanking bases on the same strand as a focal base, recognizing that a G:C base pair is not equivalent to a C:G base pair. Leading strands of oriented and ordered scaffolds in the left and the right replichores were used for this analysis. Different base substitutions occurring on the focal base of all possible 64 contexts on each replichore were called. The number of mutations was then transformed into context-specific mutation rate by dividing the total number of each context (tri-nucleotide) in one replichore (detected by a 3-bp sliding window with a 1-bp step), the number of MA lines (45) and the average number of generations passed (5,240).

### Prophage Mutations Analyses

Prophage sequences were searched for in the concatenated draft genome using the web-based PHAST (Zhou et al. 2011). After the predicted prophage sequences were confirmed by blasting against the draft genome, all *Pseudomonas* host sequences and prophages spanning two scaffolds were filtered out; mutations falling in the prophage regions were then called for the prophage base-substitution rate/spectrum analyses. There are 19 confirmed prophage regions (only one is intact, i.e., not missing any necessary prophage components); the prophage sequences are sporadically distributed across the whole genome with sizes ranging from 10.3 kb to 35.7 kb; the mean GC content is 60.27%.

### Statistics, Plotting, and Computation Environment

All statistical tests were performed in R v3.1.0 (R Development Core Team 2014).

Following one previous study (Sung, Tucker, et al. 2012), assuming that mutations follow Poisson distribution, standard error for mutation rate of an individual line is calculated by: ${\text{SE}}_{\bar{x}}=\sqrt{\frac{u_{\text{bs}}}{n\text{T}}}$, where *u*_bs_ is the base-substitution rate of the MA line, *n* is the number of sites analyzed, and *T* is the generations passed. The standard error across all MA lines is calculated by: ${\text{SE}}_{\text{pooled}}=s/\sqrt{N}$, where *s* is the average SE of the mutation rates across all MA lines, and *N* is the number of MA lines analyzed.

The odds ratio logit method (Morris and Gardner 1988) for testing whether the ratio of GC → AT transition rate $\mu_{\text{GC}\to \text{AT}}$ to AT → GC transition rate $\mu_{\text{AT}\to \text{GC}}$ equals 1 is briefly: The natural log odds ratio is approximately normally distributed and calculated by

$$\ln\frac{\mu_{\text{GC}\to \text{AT}}}{\mu_{\text{AT}\to \text{GC}}}=\ln(\frac{\pi_{\text{GC}\to \text{AT}}}{N_{\text{GC}}}/\frac{\pi_{\text{AT}\to \text{GC}}}{N_{\text{AT}}}),$$

where π_GC → AT_ is the number of transitions to AT, *N*_GC_ is the number of GC sites in the genome, π_AT → GC_ is the number of transitions to GC, and *N*_AT_ is the number of AT sites in the genome. The standard error of the log odds ratio is approximated by:

$$\begin{array}{c} \text{SE}\left(\ln\frac{\mu_{\text{GC}\to \text{AT}}}{\mu_{\text{AT}\to \text{GC}}}\right)\\ =\sqrt{\frac{1}{\pi_{\text{GC}\to \text{AT}}}+\frac{1}{\left(N_{\text{GC}}-\pi_{\text{GC}\to \text{AT}}\right)}+\frac{1}{\pi_{\text{AT}\to \text{GC}}}+\frac{1}{\left(N_{\text{AT}}-\pi_{\text{AT}\to \text{GC}}\right)}}\text{,} \end{array}$$

the one-sided *P* value is then

$$P(Z<-\frac{\left|\ln\frac{\mu_{\text{GC}\to \text{AT}}}{\mu_{\text{AT}\to \text{GC}}}\right|}{\text{SE}\left(\ln\frac{\mu_{\text{GC}\to \text{AT}}}{\mu_{\text{AT}\to \text{GC}}}\right)})\text{,}$$

where *P* is the cumulative normal probability. The paired *t*-test was performed on the natural log transformed 64 context-specific mutation rates in each replichore.

## Results

### *P. fluorescens* ATCC948 Is a Mutator Strain

Because the ATCC948 strain is highly divergent from all published *P. fluorescens* genomes, we used a de novo assembly of the MA-line sequence to determine the genotype of the progenitor strain (see Materials and Methods). Blasting the de novo assembly with the related strain SBW25 revealed a 41-bp insertion duplication in the *mutS* sequence. All final MA lines but line p20 had the *mutS* insertion, which causes a reading frame shift and premature translation termination. We further confirmed the presence of the 41-bp insertion by bidirectional Sanger sequencing of the *mutS* locus of the exceptional line p20 at an earlier time point—generation 328. Fluctuation tests using rifampicin also revealed for p20 at generation 5240 (the final MA generation) a rate of 8.72 × 10^−^^9^ mutations per generation, with 95% confidence interval (CI): 3.98 × 10^−^^9^, 14.73 × 10^−^^9^, 99-fold lower than p20 at generation 328 (8.64 × 10^−^^7^, 95% CI: 7.48 × 10^−^^7^, 9.86 × 10^−^^7^) and 80-fold lower than the original ATCC948 strain (7.05 ×10^−^^7^, 95% CI: 5.96 × 10^−^^7^, 8.21 × 10^−^^7^). Fluctuation tests performed on the *P. fluorescens* type strain (SBW25) yielded a mutation rate of 4.25 × 10^−^^9^ mutations per generation (95% CI: 2.88 × 10^−^^9^, 5.80 × 10^−^^9^), about 166× lower than that of the original ATCC948 strain. Examination of other genes involved in MMR (e.g., *mutL*, *uvrD*) found no further abnormalities. Collectively, these observations indicate that ATCC948 is a mutator strain due to a 41-bp insertion in *mutS*, and that an exact reversion of the 41-bp insertion occurred in line p20 during the course of the experiment, yielding a recovered MMR pathway and eliminating the mutator phenotype.

### Mutation Rate and Spectrum from Whole-Genome Sequencing

We identified 31,106 base-substitution mutations, across the 45 sequenced MA lines, which yields a base-substitution mutation rate of 2.34 × 10^−^^8^ (SE: 0.01 × 10^−^^8^) per site per cell division (table 1 and supplementary table S1, Supplementary Material online).

**Table 1:** Mutation Spectrum in *Pseudomonas fluorescens* ATCC948

Substitutions	Counts	Fraction
Intergenic regions	2,480	0.080
Coding regions	28,626	0.920
Synonymous	6,660	0.214
Nonsynonymous	14,903	0.479
Overlapping/gaps	7,063	0.227
Transitions	30,331	0.975
AT → GC	14,886	0.479
GC → AT	15,445	0.496
Transversions	775	0.025
AT → TA	95	0.003
AT → CG	90	0.003
GC → CG	176	0.006
GC → TA	414	0.013

Note.—“Overlapping” indicates base substitutions falling on nucleotides in overlapped reading frames, which are distinguished from nonsynonymous/synonymous mutations; fraction is the fraction of all base substitutions.

**Fig. 1.—:**
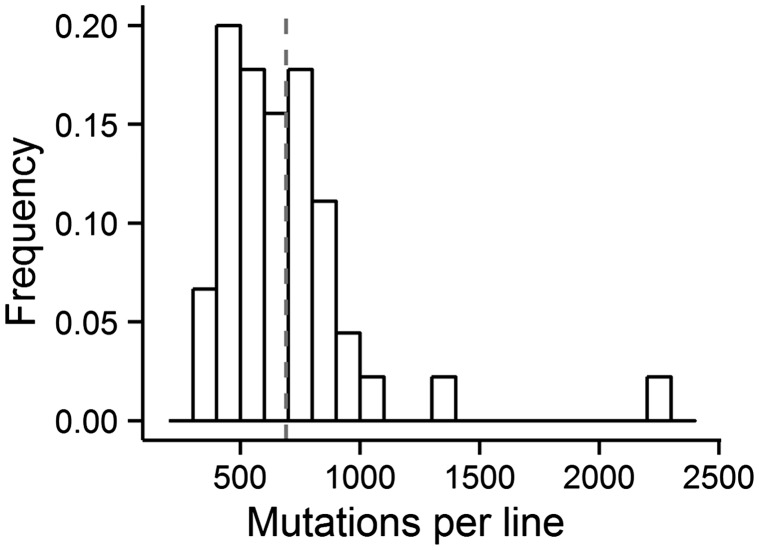
Distribution of mutations among MA lines. The histogram shows the frequency distribution of mutations, with each bin containing 100 mutations. Gray dashed line shows the mean number of mutations.

On average, 691 base substitutions accumulated in each MA line (fig. 1). We also detected a total of 2,201 small insertion/deletion events (<28 bp in length), 97.73% of which occurred in simple sequence repeat motifs (supplementary table S4, Supplementary Material online). This gives a small-indel mutation rate of 1.65 × 10^−^^9^ (SE: 0.03 × 10^−^^9^) per site per generation with an insertion bias for this mutator strain, accounting for 6.77% of all detected mutations, a ratio that is close to those observed in *Escherichia coli* (∼10%), humans (∼6%), and *Arabidopsis thaliana* (∼10%) (Lee et al. 2012; Lynch 2010b; Ossowski et al. 2010) (supplementary tables S1 and S4, Supplementary Material online). Randomly selected base substitutions and indels from each MA line were directly examined using Sanger sequencing, and each of 75 base substitutions (supplementary table S2, Supplementary Material online) and 67 indels (supplementary table S3, Supplementary Material online) were validated, supporting the accuracy of the mutation analyses.

Despite the massive mutation load, there is no indication that a significant fraction of mutations were purged from the MA lines. Examination of the functional context of each base substitution (supplementary table S5, Supplementary Material online) revealed a ratio of nonsynonymous (14,903) to synonymous (6,660) base substitutions of 2.24:1 (table 1). Given the elevated transition:transversion ratio of 39.14:1 and the codon usage of *P. fluorescens* ATCC948, the expected ratio of nonsynonymous to synonymous mutations in the absence of selection is 1.92:1 (supplementary table S6, Supplementary Material online). Although nonsynonymous sites are generally expected to be under more purifying selection than synonymous sites, the excess of nonsynonymous mutations (χ^2 ^= 55.29, df = 1, *P* = 1.04 × 10^−^^13^) is inconsistent with a strong role for selection in this MA experiment. Possibly, the deviation from the expected ratio of nonsynonymous to synonymous mutations is an artifact of errors in the annotation of this draft genome.

Approximately 92% of the *P. fluorescens* genome is coding, so we expect the ratio of base substitutions in coding regions to intergenic regions to be 10.80. However, after normalizing for GC content (see Materials and Methods), we find that the observed ratio of base substitutions in coding versus intergenic regions of this mutator strain is 11.48, significantly higher than the null expectation (χ^2 ^= 5.08, *P* = 0.024, 1 df). This elevated mutation rate in coding regions might be a reflection of transcription-induced mutation, as intergenic regions are largely untranscribed while coding regions are (Datta and Jinks-Robertson 1995; Beletskii and Bhagwat 1996).

**Fig. 2.—:**
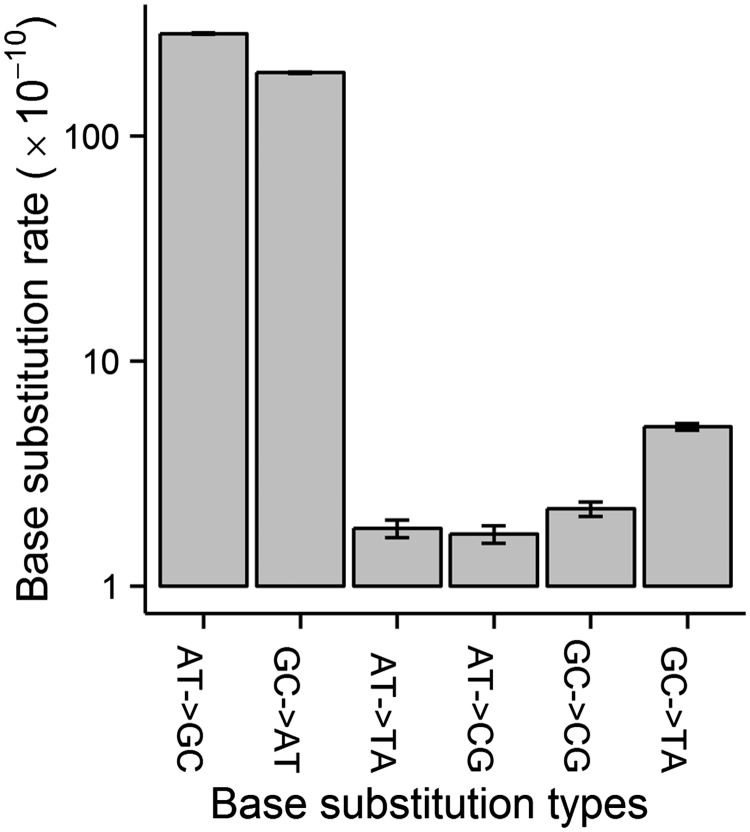
Conditional base-substitution mutation spectrum. The molecular spectrum was estimated based on all accumulated base substitutions. The base-substitution mutation rate unit is per site per generation. *y* axis is log_10_ transformed. Error bars are standard errors.

The spectrum of base substitutions in *P. fluorescens* is dominated by transitions, which arise at a rate 39× greater than that of transversions (table 1 and supplementary table S1, Supplementary Material online). The ratio of GC → AT to AT → GC transition rates is 0.67, which is significantly lower than 1 (fig. 2; supplementary table S1, Supplementary Material online) (one-sided *P* < 0.0001, odds ratio logit method).

### Mutation Rate at Different Genome Locations

**Fig. 3.—:**
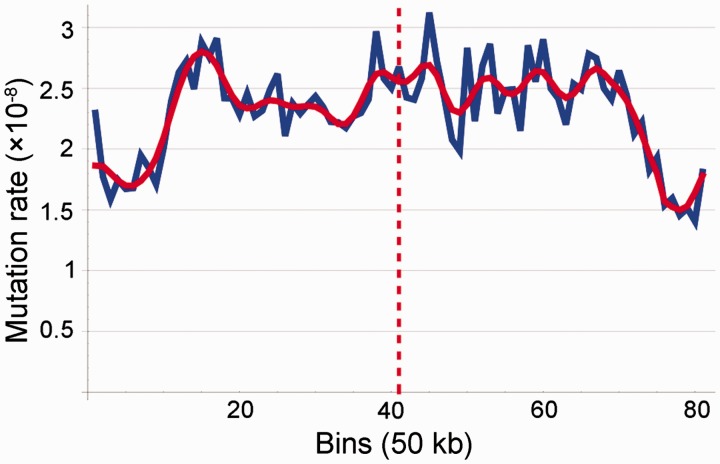
Mutation topology across the genome based on base substitutions (in 50-kb bins). The blue curve is from directly plotting data, the red curve is the signal from wavelet transformation. The replication terminus is shown by the red dashed line.

To determine whether certain genomic regions contain mutational hotspots, we separated the genome into 81 50-kb bins (approximately 71% of the draft genome) and performed a Daubechies (1990) transformation of the binned data. After correcting for differences in the nucleotide composition of each bin, we found a mostly symmetrical pattern around the replication terminus (fig. 3, bin 41), with the lowest density of mutations in bins surrounding the origin of replication (bin 1, bin 81). In order to ensure that this pattern is not an artifact of bin size, we performed the same wavelet transformation using 10-kb bins (532 bins, 93% of the draft genome) and obtained a similar result (supplementary fig. S1, Supplementary Material online).

### Nucleotide Context Influencing Mutation Rates

Previous studies (Blake et al. 1992; Hess et al. 1994; Morton 2003; Lee et al. 2012) have shown that mutations are context dependent, such that the mutation rate at a site is highly dependent on the adjacent nucleotides on the same strand. We searched for such effects in the abundant base-substitution mutations accumulated in this mutator strain (1,537 base substitutions occurring on potential methylation target sites of Dam/Dcm/EcoKI/EcoBI/HgiDII were excluded to avoid mutation rates being complicated by methylation, as methylation sites are known mutational hotspots; supplementary table S7, Supplementary Material online).

**Fig. 4.—:**
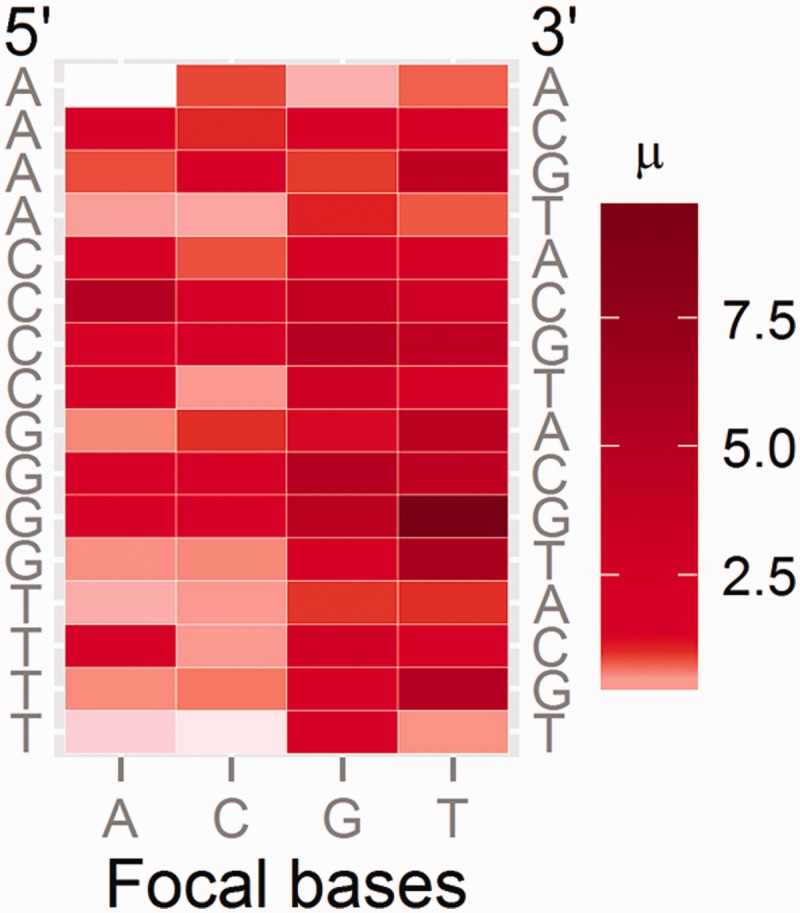
Context-dependent mutation rates. The heat map shows the mutation rates of each nucleotide context (5′-flanking nucleotide shown on the left, the focal bases at the bottom, and the 3′-flanking nucleotide on the right). µ on the scale is the base-substitution mutation rate of the focal base given a specific nucleotide context on the leading strands; numbers on the scale are in the unit of 10^−8^ per site per generation.

Separate analyses for the leading strands of the left and the right replichore indicate that the context-specific mutation rates in the two replichores are not significantly different (paired *t*-test on natural-log transformed context mutation rates, *P* = 0.31; supplementary table S8, Supplementary Material online), so we report the mean of the values for the two replichores (fig. 4; table 2; see details in each replichore in supplementary fig. S2 and table S8, Supplementary Material online). Among the 64 trinucleotide contexts, the T in the 5′-G[T → N]G-3′ triplet, where N is any nucleotide, has the highest mutation rate (mostly T → C transitions, supplementary fig. S2, Supplementary Material online), whereas the internal A in 5′-A[A → N]A-3′ has the lowest mutation rate, with the former 67.4 times higher than the latter.

**Table 2:** Mutation Rate (per Site per Generation ×10^9^) of the 64 Nucleotide Contexts, Based on the Leading Strands of Both Replichores

5′	A	C	G	T	3′
A	1.45	8.96	3.65	7.80	A
A	20.02	8.67	14.44	12.95	C
A	5.80	9.94	10.98	45.96	G
A	3.77	4.87	9.75	10.01	T
C	23.71	10.19	15.04	12.94	A
C	50.77	19.58	39.09	28.88	C
C	19.76	18.91	49.82	42.98	G
C	21.38	4.89	27.02	18.49	T
G	8.80	11.28	9.45	42.20	A
G	18.25	19.31	49.97	41.61	C
G	10.98	13.30	45.92	97.73	G
G	5.59	6.67	21.05	49.81	T
T	4.60	4.14	10.26	8.10	A
T	16.36	4.98	29.40	17.30	C
T	5.42	5.80	22.22	59.07	G
T	2.28	1.79	20.84	5.29	T

Note.—The first column is the 5′-flanking nucleotide, the four columns in the middle are the four possible focal nucleotides in the triplet, and the last column is the 3′-flanking nucleotide.

### Prophage Mutation Rate and Spectrum

There are 293 kb of prophage sequences (5% of the genome size; supplementary table S9, Supplementary Material online; 18 of 19 are incomplete, i.e., lacking necessary phage cargo genes and thus not being able to be induced to the lytic cycle) in the draft genome, and 1,466 base substitutions occurred in all the prophages. Because prophage DNA is replicated by the *P. fluorescens* machinery and the GC composition (60.27%) is close to the whole genomic level (60.71%), we expect the prophage mutation spectrum and rate to be close to that of the rest of the genome. This is the case except that the GC → AT transition rate is lower in the prophages (95% Poisson CI: 1.47 × 10^−^^8^, 1.72 × 10^−^^8^ per site per generation) than the host coding regions (95% CI: 1.91 × 10^−^^8^, 1.98 × 10^−^^8^) (supplementary table S10, Supplementary Material online), but consistent with that of the host intergenic regions (95% CI: 1.63 × 10^−^^8^, 1.84 × 10^−^^8^) (supplementary table S10, Supplementary Material online; all base-substitution types’ 95% Poisson CIs of prophage and intergenic host DNA overlap). If we assume that transcription-associated mutagenesis results in a different coding and intergenic mutation spectrum, this result suggests that prophages are not by and large transcribed in the host genome, as expected for prophages.

## Discussion

The subject strain was patented for producing 6-aminopenicillanic acid (US patent number: US3239437 A) and cholesterol esterase (US4011138 A). Because healthy bacteria strains are critical for industrial purposes, using the mutator strain *P. fluorescens* ATCC948 to produce 6-aminopenicillanic acid or cholesterol esterase may not be optimal, as many mutations will be introduced into growing cells during the mass culturing process. This can result in lower efficiency of production or even loss of the ability to produce 6-aminopenicillanic acid and cholesterol esterase, compared with MMR^+^ strains. The ATCC948 culture did show slow growth (the final MA lines grew even slower), taking approximately 2 days to grow into a 1-mm diameter colony at 24 °C, versus less than 24 h for the MMR^+^ strain SBW25 (data not shown). We propose that using an MMR rescued strain would improve the ability of *P. fluorescens* to produce 6-aminopenicillanic acid or cholesterol esterase.

Our observations of elevated mutations in coding regions are consistent with the finding in a recent *E. coli* MA study comparing the mutation spectra of MMR^+^ and MMR^−^ strains (Lee et al. 2012), suggesting that MMR may preferentially prevent errors in coding DNA, as MMR^−^ lines show a greater elevation of mutations in coding regions. Although Lee et al. (2012) were unable to establish the significance for this observation of elevated mutation rate in coding regions in their dataset, the abundant mutations accumulated in our *P. fluorescens* MA lines provide an unusual level of statistical power to examine the relationship between mutations and their functional context.

The high elevation of the transition/transversion ratio under MMR deficiency may be explained by 1) transversions may not be repaired by MMR. For instance, Liu and Chang (1988) reported that some transversions may be repaired by a pathway that is independent of the MMR system and MMR deficiency only prevents transition repair, but transversions are still repaired. 2) MMR is biased to repair transitions. This was proposed by a recent whole-genome mutation study on *E. coli* (Lee et al. 2012), in which the transition/transversion ratio was elevated by about 32 times after *mutL* was knocked out.

The symmetrical mutation pattern in MMR^−^ *P. fluorescens*, which is similar to that found in a *mutL*^−^ strain of *E. coli* (Foster et al. 2013), suggests that mutation rates are influenced by genomic position. Nonetheless, the results for prokaryotes differ from those observed in a study of MMR^−^ *Saccharomyces cerevisiae*, which found mutations to be randomly distributed across the genome and independent of replication timing (Lang et al. 2013), as did a recent study on MMR^+^ *S. cerevisiae* (Zhou et al. 2014); however, an earlier study showed a correlation between mutation rate and replication timing on chromosome VI in an MMR^+^ *S. cerevisiae* (Lang and Murray 2011)*,* consistent with our finding here. Although further investigation is needed, these different conclusions suggest that the topological dependency of MMR may differ between prokaryotes and eukaryotes, or it is variable between different chromosomes in *S. cerevisiae*.

Consistent with recent observations on *E. coli* (Lee et al. 2012), we find an elevation in mutation rate when at least one flanking nucleotide is a G or C (fig. 4; table 2). One possible explanation for this phenomenon involves base-stacking interactions: A:T or G:C pairs provide different interactions, with A:T destabilizing the DNA double helix and G:C having almost no effect (Yakovchuk et al. 2006). During DNA synthesis, if a mismatch is incorporated at a focal nucleotide flanked by two A:T pairs (i.e., when a focal A is flanked by As in 5′-AAA-3′, which has the lowest mutation rate), the distortion to the double-helix may be sufficient to disrupt the DNA polymerase’s binding to DNA and thus trigger the polymerase’s editing function. By contrast, proofreading may be less efficient when focal bases are flanked by a G or C. G/C nucleotides stabilize the pairing between strands and may thereby facilitate mismatched base pairs, as a mismatch will be less likely to destabilize the helix sufficiently to trigger proofreading. In other words, the stable pairing of G:C provides stronger docking power than does A:T pairing, thus encouraging mismatches at the focal nucleotide during DNA replication by obscuring polymerase editing function. Together with the above observation that mutation rate differs at different genomic locations, it is clear that mutations do not occur randomly, but are influenced by both local nucleotide context and genomic position.

Although the mutation results for prophages indicate that prophages are not transcribed, because most prophages in the genome of ATCC948 are incomplete, further investigation is required to determine whether the rate and spectrum of spontaneous mutations reflect that of more complete prophages or prophages in other organisms.

## Conclusions

With spontaneous mutations accumulated in the mutator strain *P. fluorescens* ATCC948 for almost 2 years, we have established the base-substitution mutation rate of this strain to be 2.34 × 10^−^^8^ per site per generation, dominated by AT → GC transitions. The large number of base substitutions in the MA lines exhibit nonrandom distribution at both the genomic and nucleotide scales. Across the whole genome, mutation rates are the lowest around the origin of replication, and higher around the replication terminus. At the nucleotide level, the two flanking bases greatly influence the base substitution mutation rate, with the mutation rate of the T in 5′-GTG-3′ being 67.4× higher than that of the A in 5′-AAA-3′. For the first time, we explored the mutation rate and spectrum of prophages, finding them to be largely similar to those of the host, especially the host intergenic regions, indicating that prophages are probably not actively transcribed during the genome MA process.

## Supplementary Material

Supplementary files S1 and S2, tables S1–S10 and figures S1 and S2 are available at *Genome Biology and Evolution* online (http://www.gbe.oxfordjournals.org/).

Supplementary Data
