# Supplementary material for: Mutation Rate, Spectrum, Topology, and Context-Dependency in the DNA Mismatch Repair-Deficient Pseudomonas fluorescens ATCC948
Source: Genome Biol Evol. 2014 Dec 23;7(1):262–71. doi: 10.1093/gbe/evu284 (PMC4316635; doi:10.1093/gbe/evu284)
Supplement: Supplementary Data [file supp_7_1_262__index.html]

Mutation Rate, Spectrum, Topology, and Context-Dependency in the DNA Mismatch Repair-Deficient Pseudomonas fluorescens ATCC948 — Supplementary Data 

# Mutation Rate, Spectrum, Topology, and Context-Dependency in the DNA Mismatch Repair-Deficient *Pseudomonas fluorescens* ATCC948

## Supplementary Data

files

**Files in this Data Supplement:**

- Supplementary Data - pdf file
- Supplementary Data - pdf file
- Supplementary Data - tif file
- Supplementary Data - tif file
- Supplementary Data - xlsx file
