# Supplementary material for: Mutation Rate, Spectrum, Topology, and Context-Dependency in the DNA Mismatch Repair-Deficient Pseudomonas fluorescens ATCC948
Source: Genome Biol Evol. 2014 Dec 23;7(1):262–71. doi: 10.1093/gbe/evu284 (PMC4316635; doi:10.1093/gbe/evu284)
Supplement: Supplementary Data [file supp_evu284_File_S1.Alignment.pdf]

Supplemental file 1. Alignments showing base pair substitutions (red, bold and underlined) in the MA lines involved in the *de novo* assembly of ATCC948-1. Each alignment shows the reference sequence from de novo assembly, sequences of MA line with the base pair substitution and the control line by Sanger sequencing.

>1:scaffold5:39543

Ref: ATCGTGCTGAGCGAGTGGTGCTATTACCTCGACCATGACGACCTGTGCCTGCTCGTCGAAC

MA: ATCGTGCTGAGCGAGTGGTGCTATTACCTC**A**ACCATGACGACCTGTGCCTGCTCGTCGAAC

Control: ATCGTGCTGAGCGAGTGGTGCTATTACCTCGACCATGACGACCTGTGCCTGCTCGTCGAAC

>1:scaffold5:41924

GGCATCGACAACTATGCGCAAAGCCGCGGGCGCGTGGCCAGCGTGGCCCAGGGCGATGGCC

GGCATCGACAACTATGCGCAAAGCCGCGGG**T**GCGTGGCCAGCGTGGCCCAGGGCGATGGCC

GGCATCGACAACTATGCGCAAAGCCGCGGGCGCGTGGCCAGCGTGGCCCAGGGCGATGGCC

>2:scaffold10:72402

ACCGCGCGGGAGATGGAACACCAGTTCAACGACTCCGGGGCCAAAGCCCTGATCTGCCTGG

ACCGCGCGGGAGATGGAACACCAGTTCAAC**A**ACTCCGGGGCCAAAGCCCTGATCTGCCTGG

ACCGCGCGGGAGATGGAACACCAGTTCAACGACTCCGGGGCCAAAGCCCTGATCTGCCTGG

>2:scaffold5:99831:De_novo

TTCTGGACCGTCAGCACCCTGCTGATGTACCCCGTGGACCTGGTCAAGGCCAGCGGCG

TTCTGGACCGTCAGCACCCTGCTGATG**C**ACCCCGTGGACCTGGTCAAGGCCAGCGGCG

TTCTGGACCGTCAGCACCCTGCTGATGTACCCCGTGGACCTGGTCAAGGCCAGCGGCG

>2:scaffold45:11165

CCTGGTCGAAGCGGACTGGGATGATGCCCCCAAGTTTCCCGAAGACTTCCGCCAGGCCGTG

CCTGGTCGAAGCGGACTGGGATGATGCCCC**T**AAGTTTCCCGAAGACTTCCGCCAGGCCGTG

CCTGGTCGAAGCGGACTGGGATGATGCCCCCAAGTTTCCCGAAGACTTCCGCCAGGCCGTG

>67:scaffold44:20683

GCCTGATAGGTGCTCCGCCCGGGTATGTCGGCTATGGCGAAGGTGGCGTCCTCACCGAGGC

GCCTGATAGGTGCTCCGCCCGGGTATGTCG**A**CTATGGCGAAGGTGGCGTCCTCACCGAGGC

GCCTGATAGGTGCTCCGCCCGGGTATGTCGGCTATGGCGAAGGTGGCGTCCTCACCGAGGC

>67:scaffold43:101644

CTGACCGGCGCGCTGGCGTTCGACCAGTTGTTCCATCTGGTAGCGATAACGAGTACCTGGG

CTGACCGGCGCGCTGGCGTTCGACCAGTTG**C**TCCATCTGGTAGCGATAACGAGTACCTGGG

CTGACCGGCGCGCTGGCGTTCGACCAGTTGTTCCATCTGGTAGCGATAACGAGTACCTGGG

>73:scaffold4:42629

ATGTTCGCGCCGCTGATAGGCCCGAGAATCTTGAACGCCACACCCGGGGTGTCTGGCACGC

ATGTTCGCGCCGCTGATAGGCCCGAGAATC**C**TGAACGCCACACCCGGGGTGTCTGGCACGC

ATGTTCGCGCCGCTGATAGGCCCGAGAATCTTGAACGCCACACCCGGGGTGTCTGGCACGC

>73:scaffold40:30646

GGGCCAGACCATCGACCACCGGGTAGACGCGGCCATCCGCAGCGACTGTCGCAAGGCCGGC

GGGCCAGACCATCGACCACCGGGTAGACGC**A**GCCATCCGCAGCGACTGTCGCAAGGCCGGC

GGGCCAGACCATCGACCACCGGGTAGACGCGGCCATCCGCAGCGACTGTCGCAAGGCCGGC

>74:scaffold67:21028

CCTTTGTCCTTCCAGTTGGCAGAAAAGATTACCGACCTGACCCCAGGTAACCTGAATCACG

CCTTTGTCCTTCCAGTTGGCAGAAAAGATT**G**CCGACCTGACCCCAGGTAACCTGAATCACG

CCTTTGTCCTTCCAGTTGGCAGAAAAGATTACCGACCTGACCCCAGGTAACCTGAATCACG

>74:scaffold68:2112

TTCTTTCTGGTTGCGTGTAGCGCCTGAAAGCCGCCGTCTGGCTGACGATCTGCCTTTTGAA

TTCTTTCTGGTTGCGTGTAGCGCCTGAAAG**T**CGCCGTCTGGCTGACGATCTGCCTTTTGAA

TTCTTTCTGGTTGCGTGTAGCGCCTGAAAGCCGCCGTCTGGCTGACGATCTGCCTTTTGAA

>74:scaffold70:1094

GATGATTTTATCGCGAACCTCGTCGCTTGGCCGATAACTCGAAGGTGTATCGGTGCGATTA

GATGATTTTATCGCGAACCTCGTCGCTTGG**T**CGATAACTCGAAGGTGTATCGGTGCGATTA

GATGATTTTATCGCGAACCTCGTCGCTTGGCCGATAACTCGAAGGTGTATCGGTGCGATTA
