# Supplementary material for: Mutation Rate, Spectrum, Topology, and Context-Dependency in the DNA Mismatch Repair-Deficient Pseudomonas fluorescens ATCC948
Source: Genome Biol Evol. 2014 Dec 23;7(1):262–71. doi: 10.1093/gbe/evu284 (PMC4316635; doi:10.1093/gbe/evu284)
Supplement: Supplementary Data [file supp_evu284_File_S2.Alignment.pdf]

Supplemental file 2. Alignments showing indels in de novo assembly libraries creating gaps. Each alignment shows the reference sequence from de novo assembly based on Illumina reads, sequences of MA line with the indel and the control line by Sanger sequencing. Indels are marked red, bold and underlined. Please note most of the flanking alignments were omitted for visualization purpose.

>1:scaffold4:9285

Ref: CAAATGTGACAGCGGGCAAGCCAGCCCCCCNNNNNNNNNNNNNNNNNNNNNN

MA: CAAATGTGACAGCGGGCAAGCCAGCCCCCC**C**TATTTTGATCTTCGCCAGTCGTA

Control: CAAATGTGACAGCGGGCAAGCCAGCCCCCC-TATTTTGATCTTCGCCAGTCGTA

>1:scaffold15:12931

GCGCTCGCGGCTGGAAGCGTTTATGGGGGG--NNNNNNNNNNNNNNNGCTTGCCAC

GCGCTCGCGGCTGGAAGCGTTTATGGGGGG**G**CTGAGAGCGCTAAGCGGCACGTCATCTCTGCGCTTCTCTTGCCGCATCCTGCTTGACGCTTGCCAC

GCGCTCGCGGCTGGAAGCGTTTATGGGGGG-CTGAGAGCGCTAAGCGGCACGTCATCTCTGCGCTTCTCTTGCCGCATCCTGCTTGACGCTTGCCAC

>67:scaffold32:82543

ATCGCCAAACATCAATTGCAGACAGGGGGG---NNNNNNNNNNACTGATAATACGGT

ATCGCCAAACATCAATTGCAGACAGGGGGG**G**AAATCTGAAGCCCGGCGCGTTCTGATTGAACTTGAAGGCTGCGACCTGGCAGGCCTGACTGATAATACGGT

ATCGCCAAACATCAATTGCAGACAGGGGGG-AAATCTGAAGCCCGGCGCGTTCTGATTGAACTTGAAGGCTGCGACCTGGCAGGCCTGACTGATAATACGGT

>67:scaffold37:206678

CGATCACGCCCAGGGCGTCAACGTGGGGGG---NNNNTCACCGGGGACATTTTCTCC

CGATCACGCCCAGGGCGTCAACGTGGGGGG**G**TGTGGCTGTCGGTGCCGACGCAGGTATCCGGGAAGGCCACGCCATCACGCACCTGGATCACCGGGGACATTTTCTCC

CGATCACGCCCAGGGCGTCAACGTGGGGGG-TGTGGCTGTCGGTGCCGACGCAGGTATCCGGGAAGGCCACGCCATCACGCACCTGGATCACCGGGGACATTTTCTCC

>67:scaffold38:13174:de_novo

CGCGGCGACCGCGATGGCCGCTGCGGTGTT---NNNNNNNNNNNNNNNTCGTCGAGGGTGCG

CGCGGCGACCGCGATGGCCGCTGCGGTGTT**T**GCGGCCAAACGGTTCGAGCAGCAAATCCAGGCCCAGTTGGCCGGTGTTGACCGCGCGGTAATCGTCGAGGGTGCG

CGCGGCGACCGCGATGGCCGCTGCGGTGTT-GCGGCCAAACGGTTCGAGCAGCAAATCCAGGCCCAGTTGGCCGGTGTTGACCGCGCGGTAATCGTCGAGGGTGCG

>73:scaffold5:119898

CCGGCAACTGGAACCAGAACATCGGCCCCC---NNNNNTGGTGAAGCCCTGGCCCAGC

CCGGCAACTGGAACCAGAACATCGGCCCCC**C**TGCACTTTGGCAGTGATGTGCACGGCAAGACCCTGGGCATCATTGGCATGGGGCGCATTGGTGAAGCCCTGGCCCAGC

CCGGCAACTGGAACCAGAACATCGGCCCCC-TGCACTTTGGCAGTGATGTGCACGGCAGGACCCAGCGCATCATTGGCATGGGGCGCATTGGTGAAGCCCTGGCCCAGC

>73:scaffold8:99634:de_novo

CACACGCAAATCCAGGGAGGCCAGCTT-GGG--
GNNNNNGGGGTAAAAAAGCATGCATCT

CACACGCAAATCCAGGGAGGCCAGCTT**G**GGGGTCCGTCAGGCGGATCTTCAGCGTGTCCAGTTCAAGGTTGTGGGTCATTGACTTG
GCCAAGGGGTAAAAAAGCATGCATCT

CACACGCAAATCCAGGGAGGCCAGCTT-GGGGTCCGTCAGGCGGATCTTCAGCGTGTCCAGTTCAAGGTTGTGGGTCATTGACTTGG
CCAAGGGGTAAAAAAGCATGCATCT
