# Supplementary figures and images for: Mutation Rate, Spectrum, Topology, and Context-Dependency in the DNA Mismatch Repair-Deficient Pseudomonas fluorescens ATCC948
Source: Genome Biol Evol. 2014 Dec 23;7(1):262–71. doi: 10.1093/gbe/evu284 (PMC4316635; doi:10.1093/gbe/evu284)

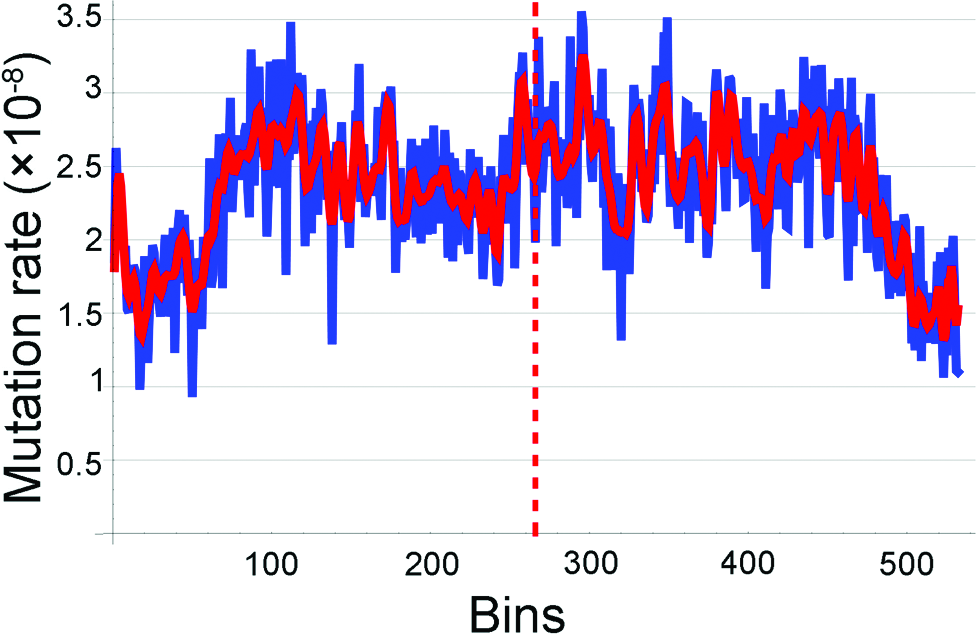

Supplement: Supplementary Data [file supp_evu284_Figure_S1.tif]

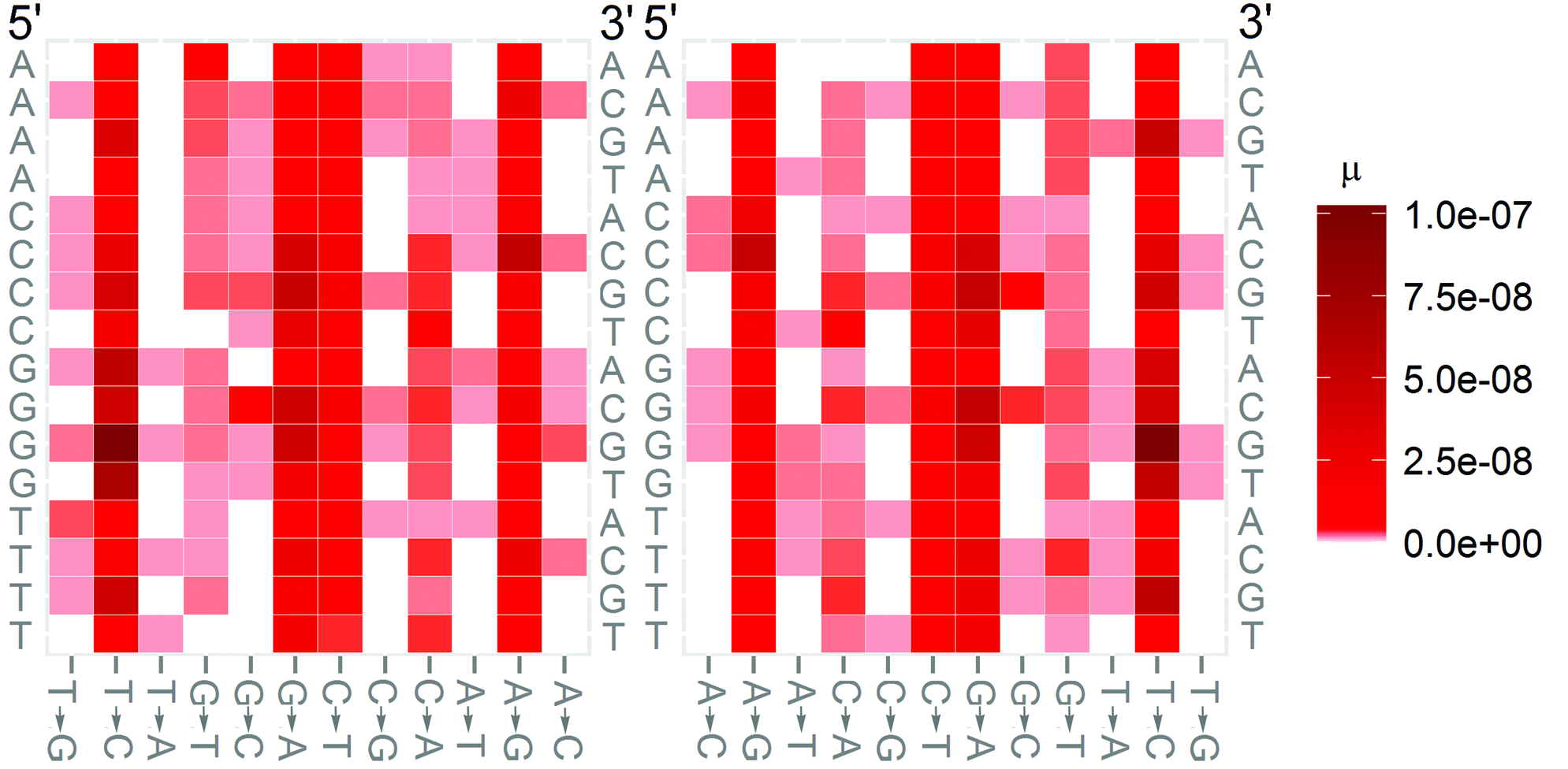

Supplement: Supplementary Data [file supp_evu284_Figure_S2.tif]
